# Microwave-Assisted Synthesis of Imidazo[4,5-*f*][1,10]phenanthroline Derivatives as Apoptosis Inducers in Chemotherapy by Stabilizing Bcl-2 G-quadruplex DNA

**DOI:** 10.3390/molecules22050829

**Published:** 2017-05-20

**Authors:** Li Li, Jie-Qiong Cao, Hui-Min Liu, Qiong Wu, Qiu-Hui Pan, Zhi-Ping Zeng, Yu-Tao Lan, Yu-Mei Li, Wen-Jie Mei, Xi-Cheng Wang, Wen-Jie Zheng

**Affiliations:** 1 The First Affiliation Hospital, Guangdong Pharmaceutical University, Guangzhou 510006, China; lili7sy@163.com (L.L.); 13570250612@163.com (H.-M.L.); 2 School of Pharmacy, Guangdong Pharmaceutical University, Guangzhou 510006, China; 13086287838@163.com (Z.-P.Z.); 15975634468@163.com (Y.-M.L.); 3 Department of Chemistry, Jinan University, Guangzhou 510006, China; caojieqiong93@163.com (J.-Q.C.); wuqiongniu.1113@163.com (Q.W.); tzhwj@jnu.edu.cn (W.-J.Z.); 4 Department of Laboratory Medicine, Shanghai Children’s Medical Center, Shanghai Jiao Tong University School of Medicine, Shanghai 200127, China; 5 School of Nursing, Guangdong Pharmaceutical University, Guangzhou 510006, China; lanyutao@gdpu.edu.cn

**Keywords:** microwave-assisted synthesis, imidazo[4,5-*f*][1,10]phenanthroline derivatives, apoptosis inducers, *bcl-2* G-quadruplex DNA

## Abstract

Herein, a series of imidazo[4,5-*f*][1,10] phenanthroline derivatives RPIP (PIP = imidazo [4,5-*f*][1,10] phenanthroline, R = NO_2_, **1**; CF_3_, **2**; Cl, **3**; OH, **4**) have been synthesized in yields of 82.3–94.7% at 100 °C under the irradiation of microwave. MTT assay has been utilized to evaluate the inhibitory activity (IC_50_) of these compounds against the growth of various tumor cells, and the results revealed that these compounds, especially **1**, exhibited excellent inhibitory activity against the growth of A549 cells with IC_50_ of 15.03 μM. Moreover, it’s also confirmed that **1** can penetrate into the membrane of tumor cells and distribute in mitochondria when observed under microscopy, resulting apoptosis of tumor cells. The further studies showed that **1** can bind to *bcl-2* G-quadruplex DNA, which demonstrated by the increase of melting point of *bcl-2* G4 DNA in the presence of **1**, as well as electronic titration and emission spectra. In a word, this kind of compound may develop as a potential apoptosis inducer in cancer chemotherapy via binding and stabilizing to the *bcl-2* G-quadruplex DNA.

## 1. Introduction

1,10-Phenanthroline derivatives with an extended π-conjugated unit of fused imidazole, which can bind to duplex DNA and G-quadruplex DNA through π-π stacking, exhibit great antitumor, anti-inflammatory and antiviral activity [1], as well as probes of DNA due to their strong fluorescence sensory properties towards acid [2]. Accumulated evidences show that this type of compounds exhibit promising inhibitory activity against various tumor cells. For example, it’s reported that imidazo[4,5-*f*][1,10]phenanthroline-dione derivatives might increase the chemosensitivity of esophageal cancer patients [3]. It’s also revealed that di-substituted phenanthroline derivatives can induce apoptosis of tumor cells through binding to human telomeric G-quadruplexes DNA and inhibit the telomerase activity significantly [4,5]. In our previous work, it has been suggested that imidazo[4,5-*f*][1,10]phenanthroimidazole derivatives showed excellent inbitory effect against the growth of breast cancer cells and lung cancer cells through binding to G-quadruplex DNA and thus blocking the replication of DNA molecules [6,7].

The *bcl-2* G-quadruplex DNA, a secondary structure of G-rich sequence in promoter of Bcl-2 gene via Hoogsteen hydrogen bond, is a key director to regulate the expression of Bcl-2, which is a crucial member of the Bcl-2 family of proteins to inhibit the apoptosis and usually over-expressed in various human tumor cells [8]. More recently, a number of small molecules like pyridostatin analog [9], metal complexes [10], quindoline derivatives [11], with high affinity to *bcl-2* G-quadruplex DNA have been reported to inhibit the transcription of Bcl-2 and thus inducing apoptosis of tumor cells. Attributed to the high ability to bind and stabilize G-qudruplex DNA, phenanthroline derivatives exhibit effectively in inducing apoptosis of various tumor cells, and it’s possible that this kind of compounds may also inhibit the growth of tumor cells through interacting with *bcl-2* G-quatruplex DNA.

**Scheme 1:**
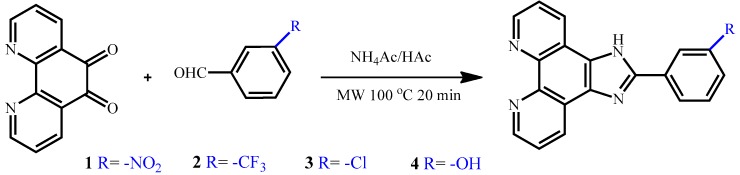
Microwave-assisted synthesis route for imidazo[4,5-*f*][1,10]phenanthroimidazole derivatives.

In this study, a series of imidazo[4,5-*f*][1,10]phenanthroline derivatives have been synthesized under microwave irradiation (Scheme 1). The anti-tumor activity of these compounds against the human lung adenocarcinoma A549 cells, human hepatocarcinoma SMMC7721 cells, and human colorectal carcinoma SW620 cells were evaluated by MTT assay. The results showed these compounds, especially **1** can effectively inhibit the growth of A549 cells. The further studies revealed that **1** could distribute in mitochondria of A549 cells, and thus inducing apoptosis of tumor cells through G1 phase arrest. Moreover, it was found that **1** exhibited moderate binding affinity to *bcl-2* G-quadruplex DNA.

## 2. Results and Discussion

### 2.1. Microwave-Assisted Synthesis of the Imidazo[4,5-f][1,10]phenanthroimidazole Derivatives

Microwave-assisted synthesis technology has been widely developed in the fields of chemical synthesis, materials science, and biotechnology [12,13]. The technique obviously shorten reaction time, while increasing yield and product purity over traditional synthesis methods, especially in organic synthesis which typically require long hours of heating in high-boiling solvents [14]. In this study, the reaction temperature rapidly reached setting value less than 60 s, and kept great stabilization and homogeneous of temperature and pressure in the whole heating process. As a result, the application of microwave irradiation has significantly increased the yields for most of the compounds to approximately 90% (Table 1), which was much higher than those with conventional methods [15].

### 2.2. Biological Activity

The synthetic imidazo[4,5-*f*][1,10]phenanthroimidazole derivatives can effectively inhibit the human lung adenocarcinoma A549 cells, human hepatocarcinoma SMMC7721 cells, and human colorectal carcinoma SW620 cells growth and proliferation, which were confirmed by MTT assay. *Cis*-platin was used as a positive control, and the inhibitory activities (IC_50_) of these complexes were listed in Table 2. It was observed that these compounds displayed great growth inhibition against various tumor cells after 72 h treatment. It’s worthy to indicate that **1** exhibited excellent inhibitory activity against A549 cells (IC_50_ = 15.03 μM) but low toxicity in normal cells. These data suggested that imidazo[4,5-*f*][1,10]phenanthroimidazole derivatives exhibited promising inhibitory activity against the growth of tumors cells.

### 2.3. Apoptosis Induction

**Figure 1:**
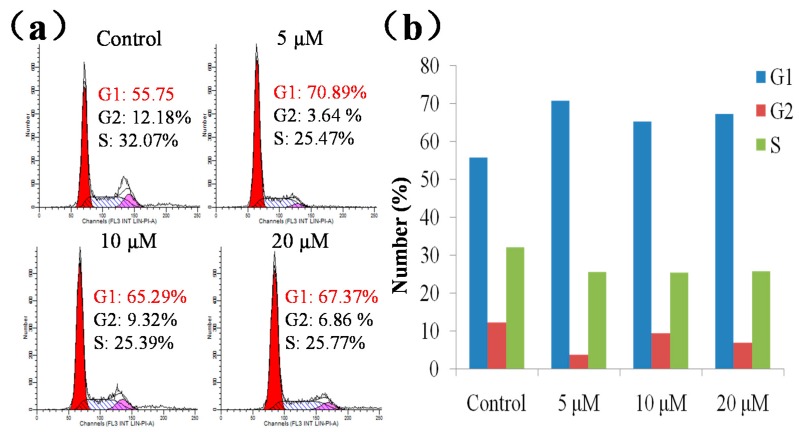
(**a**) G1-phase arrest of A549 cells induced by **1**; (**b**) Change in cell cycle distribution of A549 cells induced by **1**. A549 cells were treated with **1** (0, 5, 10, and 20 μM) for 24 h, almost 67.37% cycling cells were in the G1-phase and the sharp peak suggested that some cells were experiencing G1-phase delay or arrest.

According to the results of the MTT assay, flow cytometric analysis was carried out to further understand the underlying mechanisms of **1**. In general, apoptosis and cell cycle arrest, or a combined action of both can result in growth inhibition or death of cells [16]. After A549 cells were exposed to different concentrations of **1** (0, 5, 10, and 20 µM) for 24 h, a significant increase in G1-phase was observed, and was dosage depend (Figure 1a). At concentration of 20 µM for **1**, the ratio of G1-phase reached about 67.37%, which was about 12% higher than that of the control (55.75%) [17,18]. These data indicating that **1** may inhibit the growth of A549 cells through inducing apoptosis of tumor cells since G1 phase arrest was more related to cell apoptosis, and pro-apoptotic proteins like Bcl-2 usually plays key roles in accelerating the apoptosis of tumor cells [19,20,21,22,23].

### 2.4. Drug Distribution and Location

**Figure 2:**
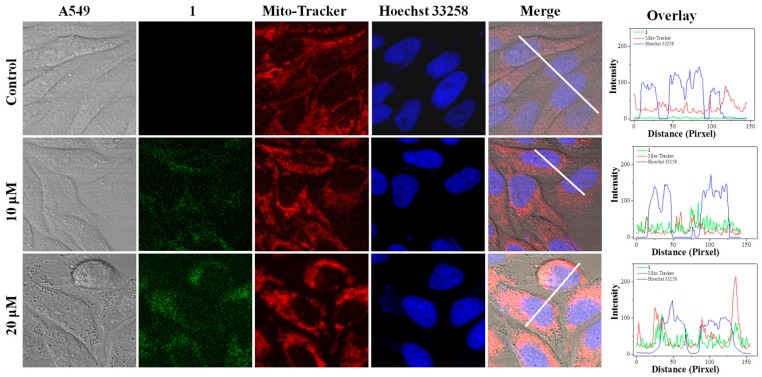
Cellular localization of **1** in A549 cells. Cells were treated with the **1** for 6 h at 37 °C [**1**] = 0, 10 and 20 μM: green, **1**; blue, Hoechst 33258; red, Mito-Tracker. The overlay data were analyzed using Image Pro Plus.

Cellular localization of **1** in A549 cells was further investigated, as shown in Figure 2. It was observed that **1** can be uptook by A549 cells and glow weak green fluorescence, a possible reason is mainly due to the absence of hypochrome nitro-group. Here, the mitochondria were marked in red by Mito-tracker, and the nuclei of A549 cells were stained blue by Hoechst 33,258. After incubated with **1** for 6 h at 37 °C, an green fluorescence was great merged with red fluorescence in the cell mitochondria, but little overlay in cell nucleus. These results indicated this compound entered into cells majorly accumulated in the mitochondria of A549 cells, which may induce the cell apoptosis through mitochondria-mediated pathway.

### 2.5. The Interaction of Bcl-2 G-quadruplex DNA with Imidazo[4,5-f][1,10]phenanthroimidazole Derivatives

#### 2.5.1. Electronic Titrations

**Figure 3:**
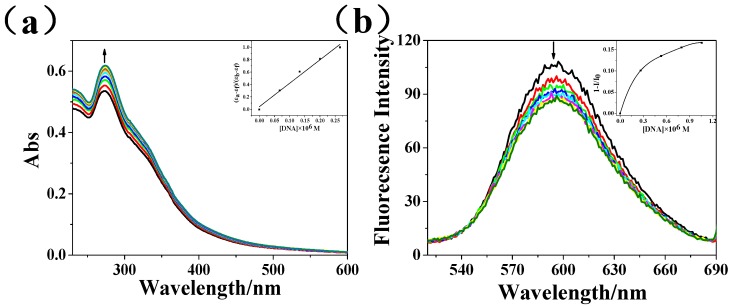
The study of the interaction between **1** with *bcl-2* G-quadruplex DNA by spectroscopic methods. (**a**) The electronic spectra of **1** in absence and in presence of *bcl-2* G-quadruplex DNA. [**1**] = 60 μM, [DNA] = 100 μM; (**b**) Emission spectra of EB and *bcl-2* G-quadruplex DNA in the incubation buffer in the absence and presence of **1**, [EB] = 16 μM, [DNA] = 2 μM.

Electronic spectra titration experiment was carried out to monitor the interaction of 1 with *bcl-2* G-quadruplex DNA. As shown in Figure 3a, the electronic spectra of **1** in Tris-HCl buffer (pH = 7.2) solution exhibited the characteristic IL (intraligand charge transfer) absorption in the range of 250–300 nm with the maximum at about 273 nm. When the DNA was gradually added to the solution of the complex, a hyperchromic effect and a bathochromic effect were observed. The hyperchromism value of **1** at the IL absorption band was about 14.1% (Δλ = 2.1 nm), which may be attributed to bases of the DNA exposed, when the ligand binding to DNA, induced external contact or to partial uncoiling of the DNA structure [24,25]. Furthermore, the value of the intrinsic binding constant (*K*_b_) for **1** was about 8.7 × 10^7^ M**^−^**^1^, calculated according to the decay of IL absorption. These data implied that **1** bound to *bcl-2* G-quadruplex DNA with high affinity in intercalating mode [26]. The interaction was further confirmed by the following fluorescence quenching experiment.

#### 2.5.2. Fluorescence Quenching

Owing to the low fluorescence of **1** in the Tris-HCl KCl buffer (pH = 7.2), fluorescence quenching of ethidium bromide (EB) and DNA was carried out. As shown in Figure 3b, when excited at 350 nm, the EB-DNA (*bcl-2* G4 DNA) emitted a strong fluorescence in the range of 500 nm to 700 nm with the maximum at about 597 nm [27]. Upon the addition of **1**, it was observed the fluorescence intensity of the solution decreased gradually. At [**1**] = 20.89 μM, the relative intensity (*I/I*_0_) for solution was about 0.83. The results implied that **1** exhibited a certain interaction with *bcl-2* G-quadruplex DNA.

#### 2.5.3. FRET Melting Point Curves

**Figure 4:**
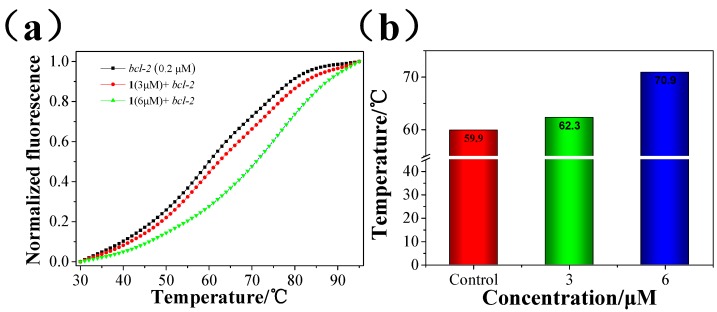
FRET melting profiles of *bcl-2* G4 DNA in the absence and in presence of **1** (**a**) ([*bcl-2* G4 DNA] = 0.2 μM) and the melting rising trend with the increasing of **1** (**b**).

To confirm the stability of the *bcl-2* G4 DNA in presence of **1**, FRET melting assay was carried out, the results were shown in Figure 4a, and the melting point of the concentration–dependent melting curves of **1** were showed in Figure 4b. Treated with 3.0 μM of **1**, the Δ*T*_m_ of *bcl-2* G4 DNA was about 2.4 °C, while the Δ*T*_m_ of *bcl-2* G4 DNA increased about 11 °C after incubated with increasing the concentration 6.0 μM of **1.** These results indicated that **1** behaved to stabilize *bcl-2* G-quadruplex DNA [28].

## 3. Experimental Section

### 3.1. Materials

All reagents were purchased from commercial suppliers and used without further purification. Solvents were dried and purified by conventional methods prior to use. Distilled water was used in all experiments. *Bcl-2* G-quadruplex DNA (5′-CGGGCGCGGGAGGAAGGGGGCGGGAGC-3′) and 5′*-*FAM*-bcl-2-*TAMRA-3′ were purchased from Sangon Biotech Co., Ltd. (Shanghai, China) *Bcl-2* G-quadruplex DNA formed a G-quadruplex conformation as literature by renaturation for 24 h at 4 °C, after denaturation for 5 min at 90 °C [29]. All aqueous solutions were prepared with doubly distilled water. The Tris-HCl buffer consisting of Tris and KCl, and the pH value was adjusted to 7.2 by HCl solution, which was applied to UV titration, Fluorescence emission titrations.

### 3.2. Instruments

The imidazo[4,5-*f*][1,10]phenanthroline derivatives were synthesized by using Anton Paar Monowave 300 microwave reactor (Anton Paar, Graz, Austria). ESI-MS spectra were obtained in methanol on Agilent 1100 ESI-MS system (Agilent, Palo Alto, CA, USA) operating at room temperature. The ^1^H-NMR and ^13^C-NMR spectra were recorded on a dimethyl-*d*_6_ sulfoxide (DMSO-*d*_6_) solution on a Bruker Avance III 500 spectrometer (Bruker, Bremen, Germany) operating at room temperature. The HPLC spectra were recorded on a Agilent 1200 high pressure liquid chromatograph (Agilent, Palo Alto, CA, USA). UV-vis absorption spectra were recorded on a Shimadzu UV-2550 spectrophotometer (Shimadzu, Tokyo, Japan). The steady-state emission spectra were recorded on a RF-5301 fluorescence spectrophotometer (Shimadzu, Tokyo, Japan). Cellular localization experiment was performed with an LSCM510 Meta Duo Scan (Carl Zeiss, Oberkochen, Germany).

### 3.3. Synthesis of Imidazole[4,5-f][1,10] phenantholine Derivatives

Imidazole[4,5-*f*][1,10]phenanthroimidazole derivatives were synthesized according to the literature procedure with some modification [30]. All of the compounds were appropriately characterized and the HPLC analysis showed the average purity of target compounds was about 97% (see Supplementary Materials).

#### 3.3.1. Synthesis of **1**

A mixture of 1,10-phenanthroline-5,6-dione (315.06 mg, 1.50 mmol), 3-nitrobenzaldehyde (339.81 mg, 2.25 mmol), ammonium acetate (4 g, 51.9 mmol) and glacial acetic acid (20 mL) was heated at 100 °C for 20 min under microwave irradiation. Then, 20 mL of water was added and the pH value was adjusted to 7.0 at room temperature. The solution was filtered and dried in vacuum to obtain a yellow precipitate, which was collected and washed with water and small amounts of ethanol. The crude product dissolved in ethanol was purified by filtration on silicagel column (60–100 mesh). ESI-MS (in MeOH, *m*/*z*): 342.1 [M + H]^+^. Anal. Calcd. for C_19_H_11_N_5_O_2_·CH_3_COOH: C, 62.84; H, 3.77; N, 17.45. Found: C, 61.66; H, 3.69; N, 18.72. ^1^H-NMR (500 MHz, DMSO) δ 9.03–9.02 (m, 1H), 9.00 (dd, *J* = 4.3, 1.7 Hz, 2H), 8.84 (dd, *J* = 8.1, 1.7 Hz, 2H), 8.64 (d, *J* = 8.0 Hz, 1H), 8.27 (dd, *J* = 8.1, 1.5 Hz, 1H), 7.83 (t, *J* = 8.0 Hz, 1H), 7.78 (dd, *J* = 8.1, 4.3 Hz, 2H). ^13^C-NMR (126 MHz, DMSO) δ 150.38 (s), 145.70 (s), 134.14 (s), 132.63 (s), 131.70 (s), 125.58 (s), 125.29 (s), 122.30 (s), HPLC purity, 98.2% at 273 nm (method A, *t*_R_ = 4.72 min).

#### 3.3.2. Synthesis of **2**

**2** was prepared using the method described above, but with 3-trifluoromethylbenzaldehyde (391.5 mg, 2.25 mmol). ESI-MS (in MeOH, *m*/*z*): 365.5 [M + H]^+^. Anal. Calcd. for C_20_H_11_F_3_N_4_·H_2_O·CH_3_CH_2_OH: C, 61.68; H, 4.47; N, 13.08. Found: C, 62.23; H, 4.26; N, 13.70. ^1^H-NMR (500 MHz, DMSO) δ 9.00 (dd, *J* = 4.3, 1.7 Hz, 2H), 8.84 (dd, *J* = 8.1, 1.6 Hz, 2H), 8.55 (s, 1H), 8.53 (d, *J* = 7.3 Hz, 1H), 7.84 (t, *J* = 3.6 Hz, 2H), 7.78 (dd, *J* = 8.1, 4.3 Hz, 2H). ^13^C-NMR (126 MHz, DMSO) δ 150.88 (s), 149.99 (s), 145.75 (s), 133.03 (s), 132.29 (s), 131.98 (s), 131.64 (s), 125.30 (s), HPLC purity, 99.3% at 273 nm (method A, *t*_R_ = 7.84 min).

#### 3.3.3. Synthesis of **3**

**3** was prepared using the method described above, but with 3-chlorobenzaldehyde (315 mg, 2.25 mmol). ESI-MS (in MeOH, *m*/*z*): 331.3 [M + H]^+^. Anal. Calcd. for C_19_H_11_ClN_4_·H_2_O·CH_3_COOH: C, 61.69; H, 4.19; N, 13.70. Found: C, 61.62; H, 3.95; N, 14.13. ^1^H-NMR (500 MHz, MeOD:CDCl_3_ = 1:1) δ 8.95–8.89 (m, 2H), 8.79 (s, 1H), 7.99 (t, *J* = 9.4 Hz, 2H), 7.62 (s, 2H), 7.57 (s, 1H), 7.29 (t, *J* = 8.7 Hz, 2H). ^13^C-NMR (126 MHz, MeOD:CDCl_3_ = 1:1) δ 149.06 (s), 144.91 (s), 141.69 (s), 131.63–131.46 (m), 131.08 (s), 127.96 (s), 124.76 (s), HPLC purity, 98.8% at 273 nm (method A, *t*_R_ = 6.30 min).

#### 3.3.4. Synthesis of **4**

**4** was prepared using the method described above, but with 3-Hydroxybenzaldehyde (274.59 mg, 2.25 mmol). ESI-MS (in MeOH, *m*/*z*): 313.1 [M + H]^+^. Anal. Calcd for C_19_H_12_N_4_O·H_2_O·CH_3_CH_2_OH: C, 67.01; H, 5.36; N, 14.88. Found: C, 67.06; H, 5.20; N, 15.25. ^1^H-NMR (500 MHz, DMSO) δ 9.02 (dd, *J* = 4.3, 1.7 Hz, 2H), 8.91 (dd, *J* = 8.1, 1.7 Hz, 2H), 7.81 (dd, *J* = 8.1, 4.3 Hz, 2H), 7.73 (dd, *J* = 6.0, 3.8 Hz, 1H), 7.72–7.69 (m, 1H), 7.39 (t, *J* = 7.9 Hz, 1H), 6.91 (ddd, *J* = 8.1, 2.4, 0.8 Hz, 1H). ^13^C-NMR (126 MHz, DMSO) δ 152.95 (s), 149.75 (s), 145.55 (s), 133.43 (s), 132.07 (s), 131.68 (s), 125.30 (s), 119.07 (s), 118.72 (s), 115.16 (s), HPLC purity, 93.2% at 273 nm (method A, *t*_R_ = 4.17 min).

### 3.4. Cell Lines, Cell Culture and MTT Assay

Human cancer cell lines, human lung adenocarcinoma A549 cells, human hepatocarcinoma SMMC7721 cells, human colorectal carcinoma SW620 cells and HaCaT cells were purchased from American Type Culture Collection (ATCC, Manassas, VA, USA). All cell lines were maintained in Dulbecco’s Modified Eagle Medium (DMEM) media supplemented with fetal bovine serum (10%), penicillin (100 units/mL), and streptomycin (50 units/mL) at 37 °C in a CO_2_ incubator (95% relative humidity, 5% CO_2_).

Cell viability was confirmed by measuring the ability of cells to transform 3-(4,5-dimethylthia-zol-2-yl)-2,5-diphenyltetrazolium bromide (MTT) to a purple formazan dye [31]. Cells were seeded in 96-well tissue culture plates (3 × 10^3^ cells per well) for 24 h. The cells were then incubated with the tested compounds at different concentrations for 72 h. After incubation, 20 µL per well of MTT solution (5 mg/mL in phosphate buffered saline, PBS) was added, followed by incubation for a further 5 h. The medium was aspirated and replaced with 150 µL/well of DMSO to dissolve the formazan salt formed. The colour intensity, which reflects the cell growth condition, was measured at 570 nm using a microplate spectrophotometer (SpectroAmaxTM250, BioTek Instruments, Inc., Winooski, VT, USA).

### 3.5. Flow Cytometric Analysis

Cells were seeded in six-well tissue culture plates (1 × 10^5^ cells per well), and the cell cycle arrest was analyzed by flow cytometry as previously described [32]. After incubating with different concentrations of **1** (0, 5, 10, and 20 μM) for 72 h, cells were trypsinized, washed with PBS, and fixed with 70% ethanol overnight at 4 °C. The fixed cells were washed with PBS and stained with propidium iodide (PI) for 15 min in the dark, then the cell cycle arrest was analyzed with an Epics XL-MCL flow cytometer (Beckman Coulter, Miami, FL, USA).

### 3.6. Cellular Localization

Cells were cultured in DMEM medium supplemented with 10% fetal bovine serum (FBS) at 37 °C under 5% CO_2_. Cells in complete growth medium at 5 × 10^4^ cells per mL were incubated for 6 h at 37 °C. Cells were treated with **1** in DMEM for 6 h at 37 °C under 5% CO_2_. Then, cells were stained with Mito-tracker and Hoechst 33,258 for another 20 min, and finally, luminescence imaging was carried out by confocal laser scanning microscope.

### 3.7. Electronic Absorption Measurements

Electronic absorption spectra were recorded on a Shimadzu UV-2550 spectrophotometer using 1 cm path length quartz cuvettes (3 mL). The absorption titration of the target complex in Tris-HCl buffer was performed by using a fixed complex concentration to which increments of the DNA stock solution were added. The concentration of the complex solution was 20 μM and *bcl-2* G4 DNA was added by degrees. Complex-DNA solutions were allowed to incubate for 3 min before the absorption spectra were recorded.

### 3.8. Fluorescence Quenching Measurements

Fluorescence spectroscopy measurements were performed on a RF-5301 fluorescence spectrophotometer using a 1 cm path length quartz cell. Fluorescence quenching of the EB + *bcl-2* G4 DNA system can be used for a compound having an affinity to DNA in spite of its binding mode, and only measures the ability of the compound to affect the EB fluorescence intensities in the EB + *bcl-2* G4 DNA system. The titration processes were repeated until there was no apparent change in the spectra for at least three titrations, indicating the achievement of the binding saturation [33].

### 3.9. FRET Melting Assays

The fluorescent labeled oligonucleotide, *bcl-2* G-quadruplex DNA (5′-FAM-CGGGCGCGGGAGGAAGGGGGCGGGAGC-TAMRA-3′, FAM: carboxyfluorescein, TAMRA: 6-carboxy tetramethylrhodamine) used as the FRET probe was diluted in TrisHCl buffer and then annealed by being heated to 90 °C for 5 min, followed by slowly cooling to room temperature [29]. Ds26 duplex DNA (CAATCGGATCGAATTCGATCCGATTG) was competitive binders to evaluate the selective binding ability of **1** with *bcl-2* G4 DNA. Fluorescence melting curves were determined with a Bio-RadiQ5 realtime PCR detection system (Bio-Rad, Berkeley, CA, USA), by using a total reaction volume of 25 mL, with labeled oligonucleotide (1 μM) and different concentrations of complex in Tris-HCl buffer [34]. A constant temperature was maintained for 30 s prior to each reading to ensure a stable value. Final analysis of the data was carried out by using Origin7.5 (Origin Lab, Northampton, MA, USA).

## 4. Conclusions

**Figure 5:**
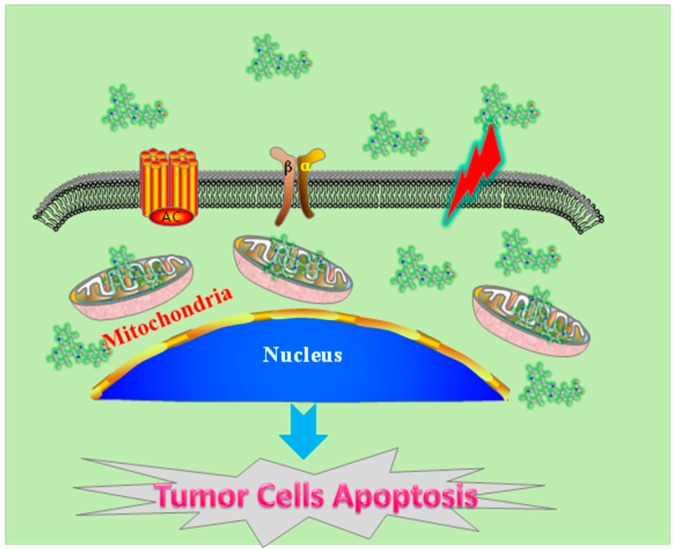
Tumor cells apoptosis induced by imidazole[4,5-*f*][1,10]phenanthroimidazole derivatives related to mitochondria-mediated pathway.

In summary, a series of imidazole[4,5-*f*][1,10]phenanthroimidazole derivatives have been synthesized by using microwave-assisted synthesis technology, with high yields of approximately 90%, which can rapid rising to the required temperature, and kept almost no change during the whole process. The results of MTT assay showed these complexes can block the growth of tumor cells, especially **1**, exhibited excellent antitumor activity against A549 cells through inducing apoptosis of the cells. The further study displayed **1** can distribute at mitochondria, and inhibit tumor cells at G1 phase then induce apoptosis. Furthermore, this compound can bind to the *bcl-2* G-quadruplex DNA. Take together, the results suggested that the target compound could act as a potential apoptosis inducer mediated-mitochondria through binding to the *bcl-2* G-quadruplex DNA in cancer chemotherapy (Figure 5).

## Figures and Tables

**Table 1:** The yields of the target complexes by microwave-assisted synthesis method.

Comp.	Microwave-Assisted		
	Temperature/°C	Time/min	Yield%
**1**	100	20	91.3%
**2**	100	20	82.3%
**3**	100	20	94.7%
**4**	100	20	89.7%

**Table 2:** The inhibitory effect IC_50_ (μM) of the target complexes and *cis*-platin on human cancer cells and normal cells at 72 h.

Comp.	IC_50_ (μM)			
	A549	SW620	SMMC-7721	HaCaT
**1**	15.03 ± 1.01	26.48 ± 0.59	17.51 ± 1.84	27.26 ± 1.49
**2**	13.79 ± 0.53	27.23 ± 0.53	12.93 ± 0.51	17.48 ± 0.58
**3**	14.27 ± 0.21	16.02 ± 0.29	12.64 ± 0.35	13.37 ± 0.56
**4**	3.00 ± 0.14	16.52 ± 0.28	24.22 ± 2.28	13.94 ± 0.64
***cis*-platin**	32.01 ± 4.66	6.29 ± 0.17	11.66 ± 1.45	18.48 ± 1.64

## Supplementary Materials

Supplementary materials are available online.
