# Supplementary material for: Microwave-Assisted Synthesis of Imidazo[4,5-f][1,10]phenanthroline Derivatives as Apoptosis Inducers in Chemotherapy by Stabilizing Bcl-2 G-quadruplex DNA
Source: Molecules. 2017 May 20;22(5):829. doi: 10.3390/molecules22050829 (PMC6154642; doi:10.3390/molecules22050829)
Supplement: Supplementary file 1 [file molecules-22-00829-s001.pdf]

Supporting Information for Microwave-assisted Synthesis of Imidazole [4,5-*f*][1,10]phenanthroline Derivatives as Apoptosis Inducers in Chemotherapy by Stabilizing Bcl-2 G-quadruplex DNA

Li Li[1,#], Jie-Qiong Cao[2,#], Qiong-Wu[2], Qiu-Hui Pan[3,*], Zhi-Ping Zeng[1], Yu-Tao Lan[4,*], Yu-Mei Li[1], Wen-Jie Mei[1,*], Wen-Jie Zheng[2]

1. The HPLC analysis of phenanthroimidazole derivatives

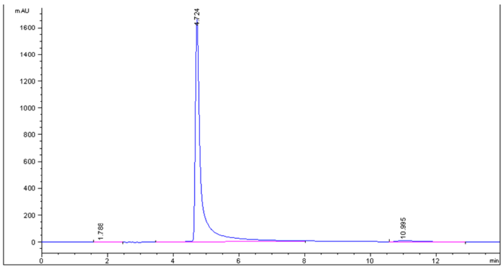

a

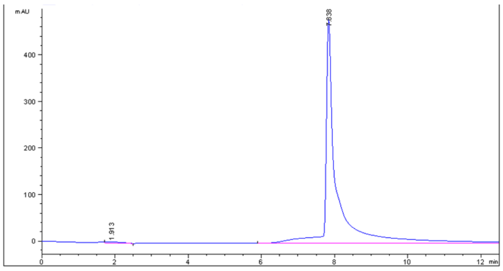

b

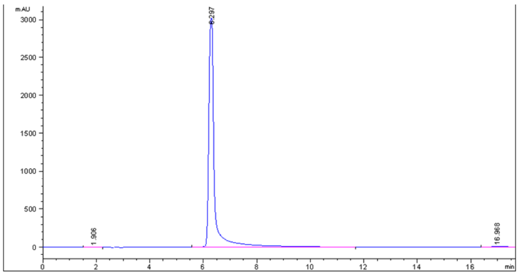

c

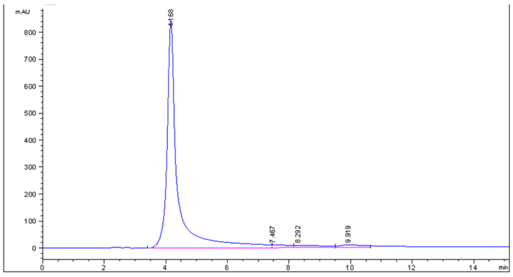

d

Figure S1. The HPLC spectras (273 nm) of phenanthroimidazole derivatives **1-4(a-b)** in the mixed solution of CH_3CN and H_2O (the volume ratio is 1:1).

Table S1.The results of HPLC analysis of phenanthroimidazole derivatives **1-4.**

Comp.	tR	A	A%
**1**	4.724	19770.5	98.247
**2**	7.838	9797.7	99.268
**3**	6.297	43882.9	98.790
**4**	4.168	19837.1	93.232

2. The results of elemental analysis of phenanthroimidazole derivatives 1-4

DATE 10 01 16 TIME 15 56 18 OPERATOR ID 01

RUN 1 ID 1 WEIGHT 3.740

SIGNALS

	ZR 24756
CARBON 61.66%	NR 28576
HYDROGEN 3.69%	CR 62540
NITROGEN 18.72%	HR 65039

DATE 10 01 16 TIME 16 01 26 OPERATOR ID 01

RUN 2 ID 2 WEIGHT 2.928

SIGNALS

	ZR 24764
CARBON 62.23%	NR 27050
HYDROGEN 4.26%	CR 53868
NITROGEN 13.70%	HR 56166

DATE 10 01 16 TIME 16 06 35 OPERATOR ID 01

RUN 3 ID 3 WEIGHT 2.999

SIGNALS

	ZR 24769
CARBON 61.62%	NR 27170
HYDROGEN 3.95%	CR 54371
NITROGEN 14.13%	HR 56572

DATE 10 01 16 TIME 16 11 43 OPERATOR ID 01

RUN 4 ID 4 WEIGHT 2.001

SIGNALS

	ZR 24772
CARBON 67.06%	NR 26565
HYDROGEN 5.20%	CR 46298
NITROGEN 15.25%	HR 48283

Figure S2. The data of elemental analysis of phenanthroimidazole derivatives **1-4.**

3. The ESI-MS spectras of phenanthroimidazole derivatives 1-4

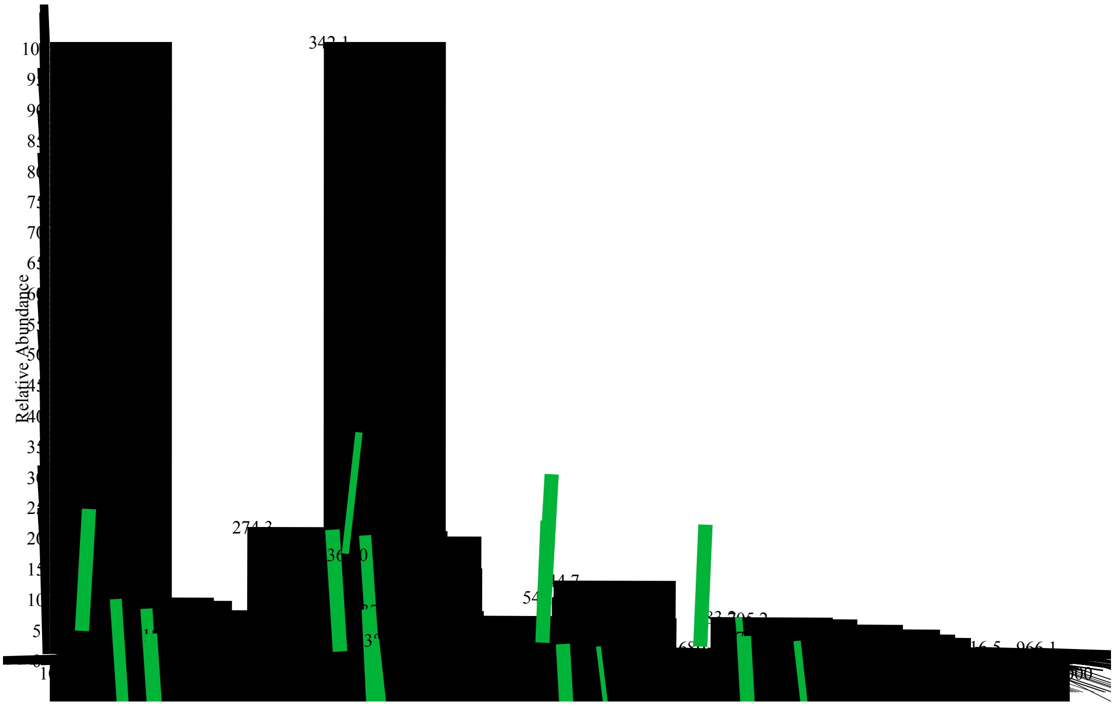

Figure S3. The ESI-MS spectra of phenanthroimidazole derivative **1**.

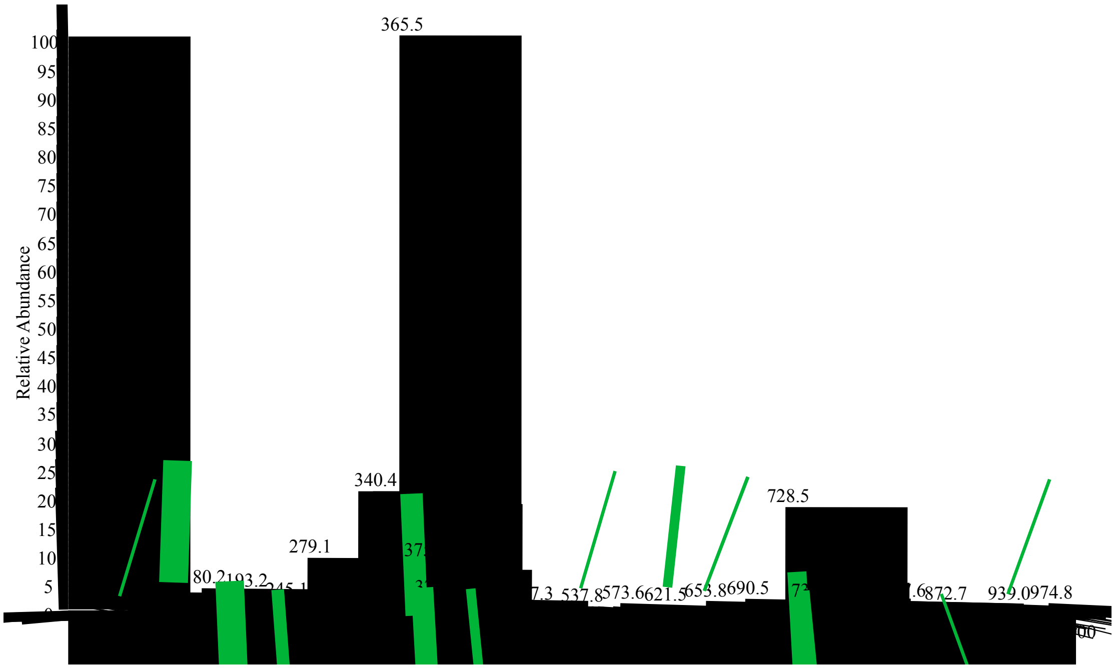

Figure S4. The ESI-MS spectra of phenanthroimidazole derivative **2**.

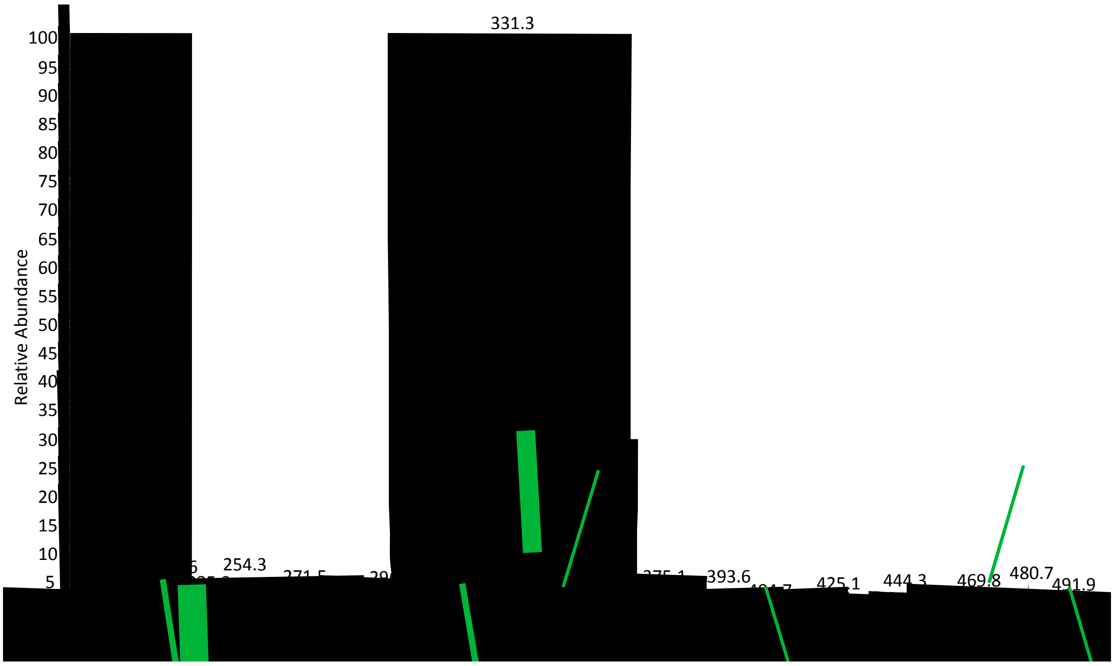

Figure S5. The ESI-MS spectra of phenanthroimidazole derivative **3**.

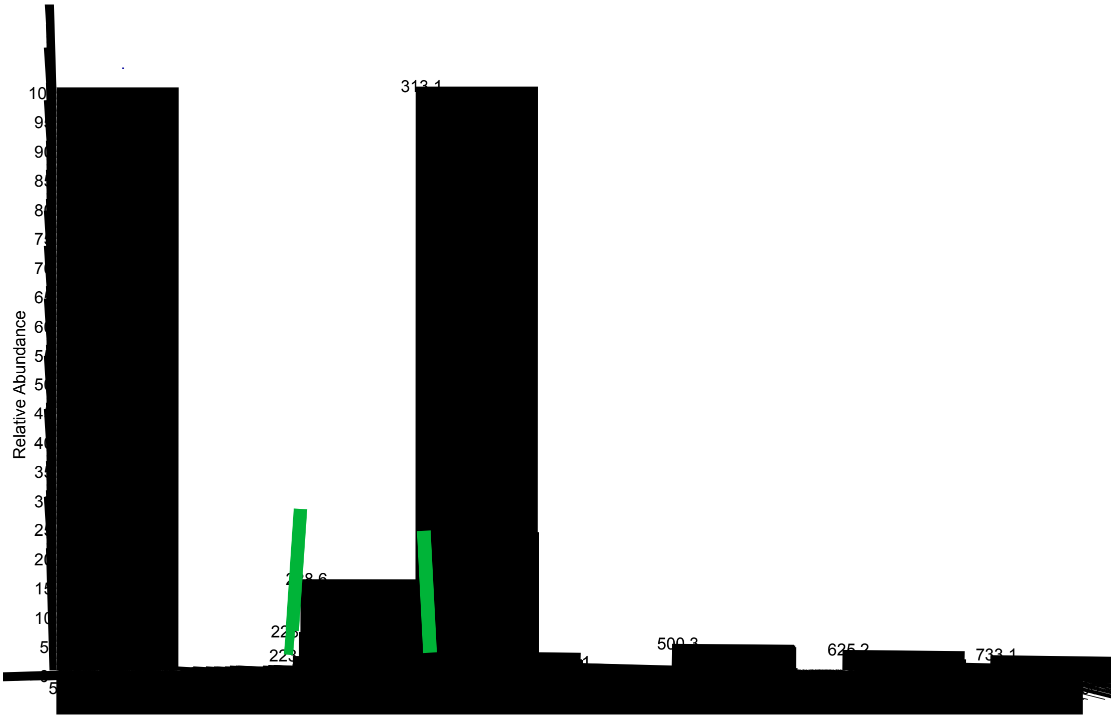

Figure S6. The ESI-MS spectra of phenanthroimidazole derivative **4**.

3. The ^{1}H NMR spectras of phenanthroimidazole derivatives

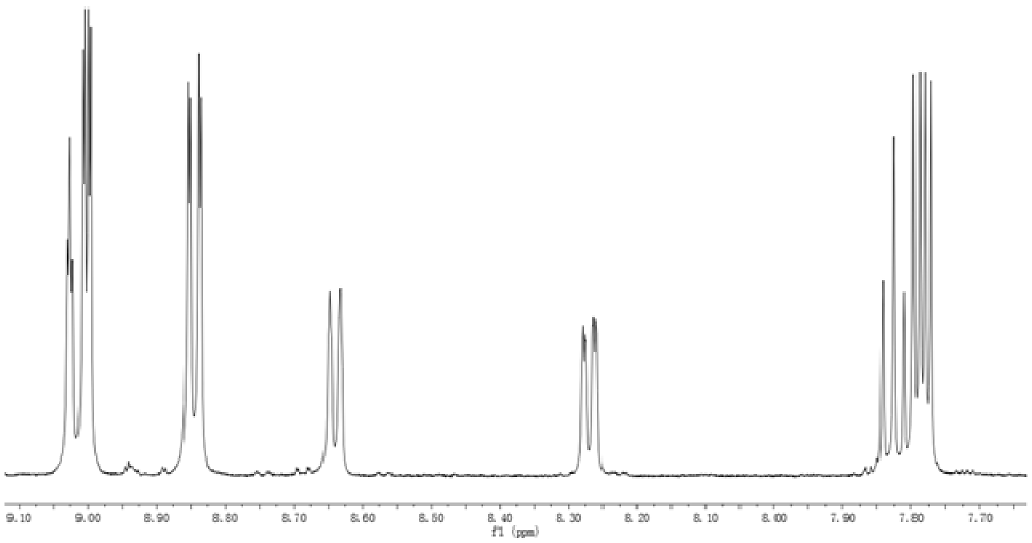

Figure S7. The ^{1}H NMR spectra of phenanthroimidazole derivative **1**.

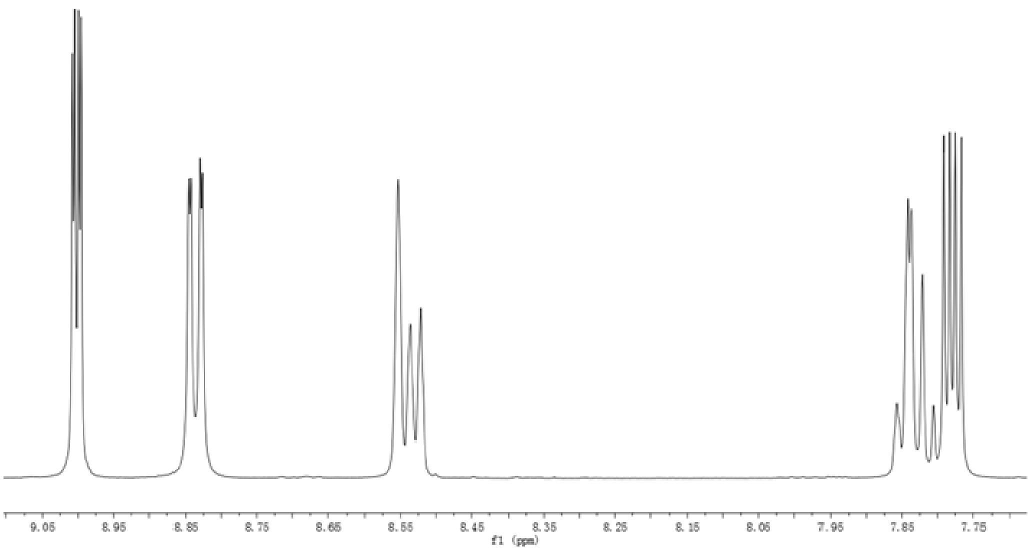

Figure S8. The ^{1}H NMR spectra of phenanthroimidazole derivative **2**.

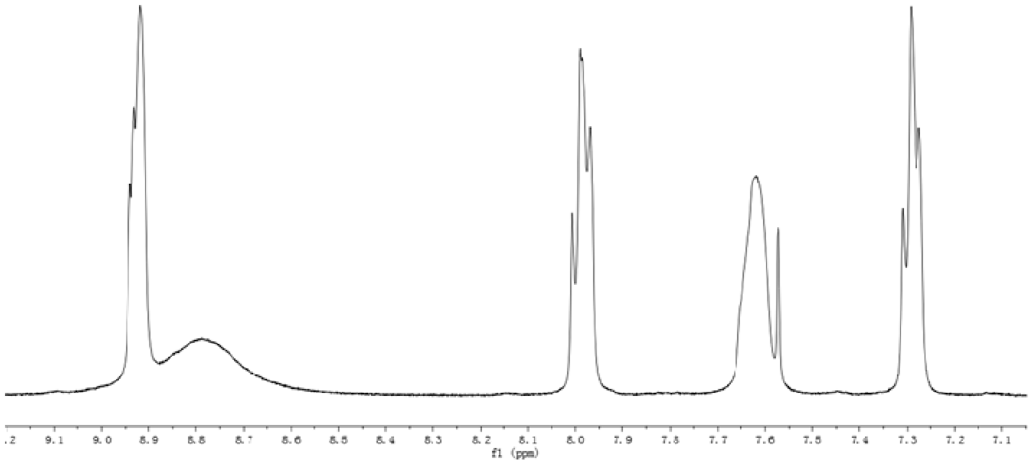

Figure S9. The ^{1}H NMR spectra of phenanthroimidazole derivative **3**.

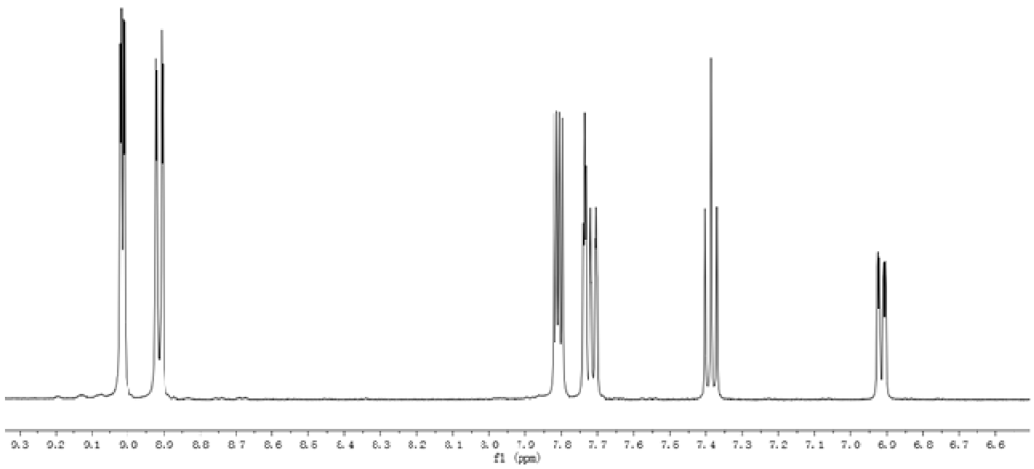

Figure S10. The ^{1}H NMR spectra of phenanthroimidazole derivative **4**.

4. The ^{13}C NMR spectras of phenanthroimidazole derivatives

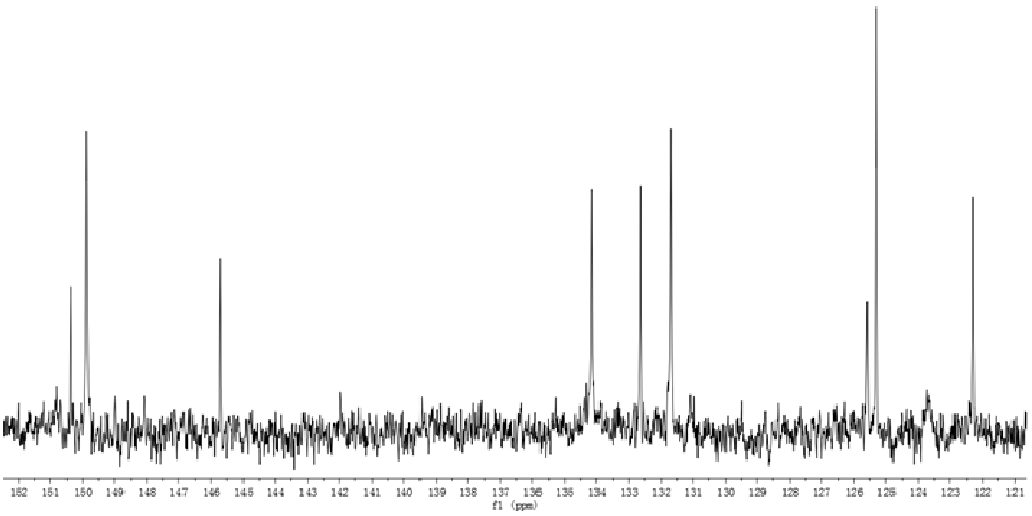

Figure S11. The ^{13}C NMR spectra of phenanthroimidazole derivative **1**.

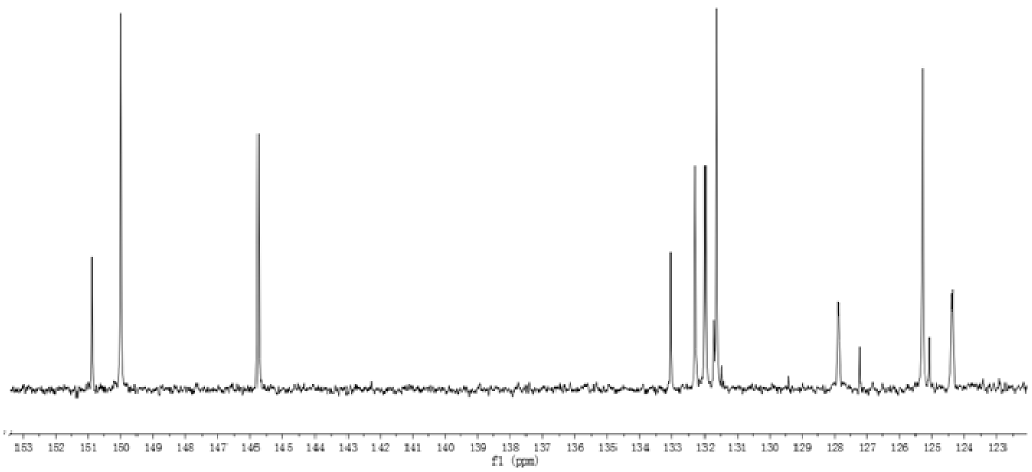

Figure S12. The ^{13}C NMR spectra of phenanthroimidazole derivative **2**.

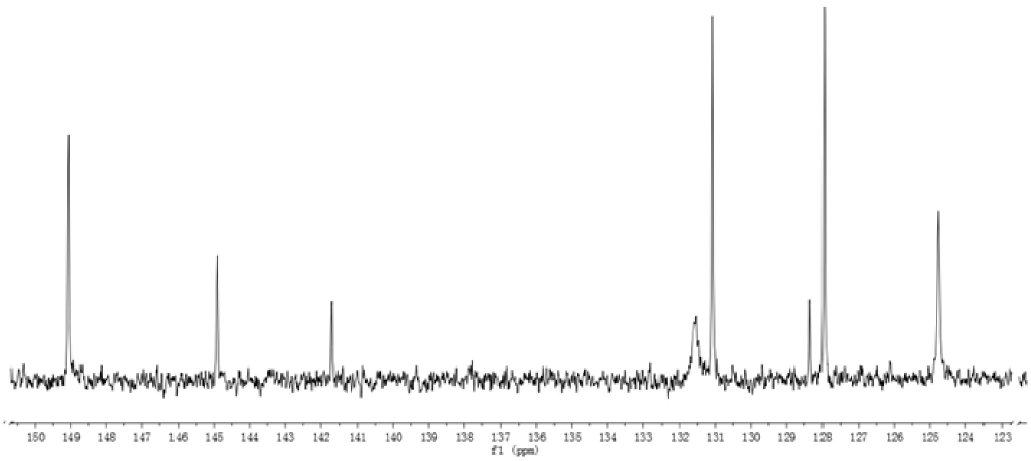

Figure S13. The ^{13}C NMR spectra of phenanthroimidazole derivative **3**.

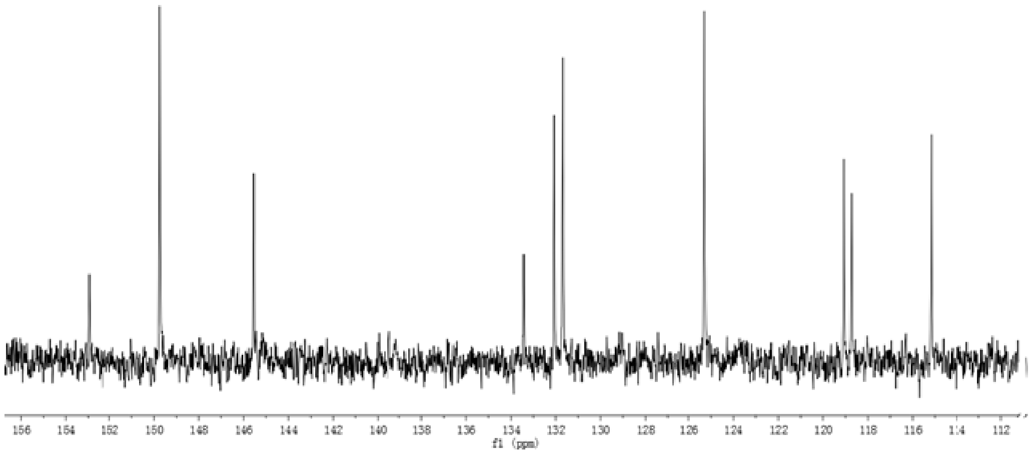

Figure S14. The ^{13}C NMR spectra of phenanthroimidazole derivative **4**.
